# Two-photon excitation fluorescent spectral and decay properties of retrograde neuronal tracer Fluoro-Gold

**DOI:** 10.1038/s41598-021-97562-3

**Published:** 2021-09-10

**Authors:** Matthew Q. Miller, Iván Coto Hernández, Jenu V. Chacko, Steven Minderler, Nate Jowett

**Affiliations:** 1 Surgical Photonics and Engineering Laboratory, Massachusetts Eye and Ear, Harvard Medical School, 243 Charles Street, Boston, MA 02114 USA; 2 Laboratory for Optical and Computational Instrumentation, University of Wisconsin, Madison, WI USA; 3 Department of Otolaryngology/Head and Neck Surgery, University of North Carolina Health Care, Chapel Hill, NC USA

**Keywords:** Cellular neuroscience, Neural circuits, Peripheral nervous system, Nervous system, Multiphoton microscopy

## Abstract

Fluoro-Gold is a fluorescent neuronal tracer suitable for targeted deep imaging of the nervous system. Widefield fluorescence microscopy enables visualization of Fluoro-Gold, but lacks depth discrimination. Though scanning laser confocal microscopy yields volumetric data, imaging depth is limited, and optimal single-photon excitation of Fluoro-Gold requires an unconventional ultraviolet excitation line. Two-photon excitation microscopy employs ultrafast pulsed infrared lasers to image fluorophores at high-resolution at unparalleled depths in opaque tissue. Deep imaging of Fluoro-Gold-labeled neurons carries potential to advance understanding of the central and peripheral nervous systems, yet its two-photon spectral and temporal properties remain uncharacterized. Herein, we report the two-photon excitation spectrum of Fluoro-Gold between 720 and 990 nm, and its fluorescence decay rate in aqueous solution and murine brainstem tissue. We demonstrate unprecedented imaging depth of whole-mounted murine brainstem via two-photon excitation microscopy of Fluoro-Gold labeled facial motor nuclei. Optimal two-photon excitation of Fluoro-Gold within microscope tuning range occurred at 720 nm, while maximum lifetime contrast was observed at 760 nm with mean fluorescence lifetime of 1.4 ns. Whole-mount brainstem explants were readily imaged to depths in excess of 450 µm via immersion in refractive-index matching solution.

## Introduction

Retrograde neuronal labeling permits study of complex neural circuits, neuropathology, and nerve regeneration^1,2^. In 1873, Golgi employed silver staining to visualize nervous tissue under light microscopy^3^. Ramón y Cajal optimized this technique in his pioneering neuroanatomical studies that established the neuron doctrine^3,4^. Weiss and Hiscoe described anterograde axonal transport in 1948^5^. In 1971, Kristensson and Olsson demonstrated retrograde axonal transport by injecting horseradish peroxidase (HRP) into murine gastrocnemius muscle with subsequent observation of tracer in spinal cord sections^6^. Evans blue was the first fluorescent dye used for retrograde neuronal labeling; many others have since been reported^1,7–12^. Fluorescent dyes allow high-fidelity neuronal labeling without resource-intensive immunohistochemical techniques.

Schmued and Fallon first described Fluoro-Gold (FG) in 1986, noting its intense fluorescence, specific labeling of damaged axons, and resistance to photobleaching^13^. Hydroxystilbamidine (OHSA) is the active fluorophore in FG, an amidine similar to DAPI, True blue, and other substances that undergo retrograde transport^14^. After axonal uptake, FG accumulates in acidic lysosomes and endosomes and is transported to the cell body where it labels cytoplasm and dendrites^13,14^. It produces a broad fluorescence emission spectrum, with an intense yellow peak at neutral pH that is blue-shifted in acidic environments^13^.

FG can be used to label neuronal cell bodies of the central and peripheral nervous systems, and shows minimal neurotoxicity at low concentrations (2–5%)^10,13,15–19^. FG is toxic to sensory and motor neurons with prolonged exposure and, similar to other retrograde dyes, induces reversible motor and sensory deficits^20,21^. Within the peripheral nervous system, FG may be employed for labeling intact motor neurons via intramuscular injection, or axotomized motor and sensory neurons via immersion of proximal nerve stumps, using conduit reservoir or crystal application techniques^1,9,13,14,22^. Hayashi et al. demonstrated conduit reservoir delivery of FG labeled the greatest number of neurons^1^. FG is compatible with immunohistochemistry (IHC) and tissue clearing techniques^23–25^.

Optimal single-photon excitation of FG is achieved using ultraviolet light. Imaging of FG-labeled specimens is typically performed using widefield fluorescence microscopy, with standard DAPI/Hoechst filter sets yielding narrow-band 365 nm excitation and long-pass filters providing broadband detection^1,9,13,14,26,27^. However, widefield imaging lacks depth discrimination, preventing optical sectioning and high-resolution three-dimensional (3D) imaging. Confocal microscope short-wavelength excitation lasers at 405 nm are not suitable for excitation of FG^14^. Secondary tagging of FG by immunofluorescence may be employed to visualize FG using visible excitation lines of commercial confocal microscopes, though this approach is resource-intensive^28,29^.

Two-photon excitation microscopy (2PEM) is an alternative to confocal microscopy for volumetric imaging of biological tissues. Maria Goeppert-Mayer characterized the theoretical basis for 2PEM in 1931^30^. Six decades later, Denk et al. first demonstrated the technique^31^. Two-photon excitation (2PE) employs near-infrared (NIR) ultrafast laser pulses to achieve simultaneous absorption of two low-energy photons by a fluorophore typically excited by a single higher-energy photon. Multiphoton NIR excitation is possible for manifold fluorophores, including many which are not suitably excited using visible light^32,33^. Use of NIR excitation in the optical window of biological tissue in 2PEM permits microscale resolution of labeled structures in highly-scattering thick tissues^34^. Owing to the quadratic dependence of fluorescence signal on excitation intensity in 2PEM, out-of-plane fluorescence is largely avoided, providing enhanced axial resolution compared to scanning laser confocal microscopy^31^. Under ideal conditions, confocal microscopy can image tissues at depths up to 100 µm, while depths up to 1 mm have been reported with 2PEM^35–38^. Though the 2PE spectral properties of many fluorescent dyes have been documented, the 2PEM excitation spectra of FG have not been heretofore characterized^39^.

Due to FG’s broad fluorescent emission spectrum, spectral overlap with other fluorophores is common and can hinder multicolor imaging. Several approaches may be employed for multicolor imaging of FG- labeled specimens. In single-photon imaging, UV excitation of FG may enable its separation from fluorophores not excited by UV light^15,40^. Fluorescence lifetime imaging microscopy (FLIM) obtains images based on fluorophore decay rate in lieu of fluorescent intensity, yielding means to resolve fluorescent labels with overlapping absorption and emission spectra^41–43^. FLIM necessitates the use of short-pulse excitation lasers, and may be implemented using single or multiphoton excitation techniques. Ultrafast pulsed lasers used for 2PEM are well-suited for FLIM; such microscope systems can readily be upgraded to enable FLIM^41^. A priori knowledge of specific decay rates of various fluorophores enables the selection of optimal candidates for multilabel experiments. Heretofore, the decay rate of FG has not been characterized.

Herein, we characterize the 2PE spectra and fluorescent lifetime distribution of FG in aqueous solution and murine facial motor nuclei. We then demonstrate the utility of 2PEM for high-throughput deep volumetric imaging of FG-labeled mammalian neurons.

## Results

**Figure 1:**
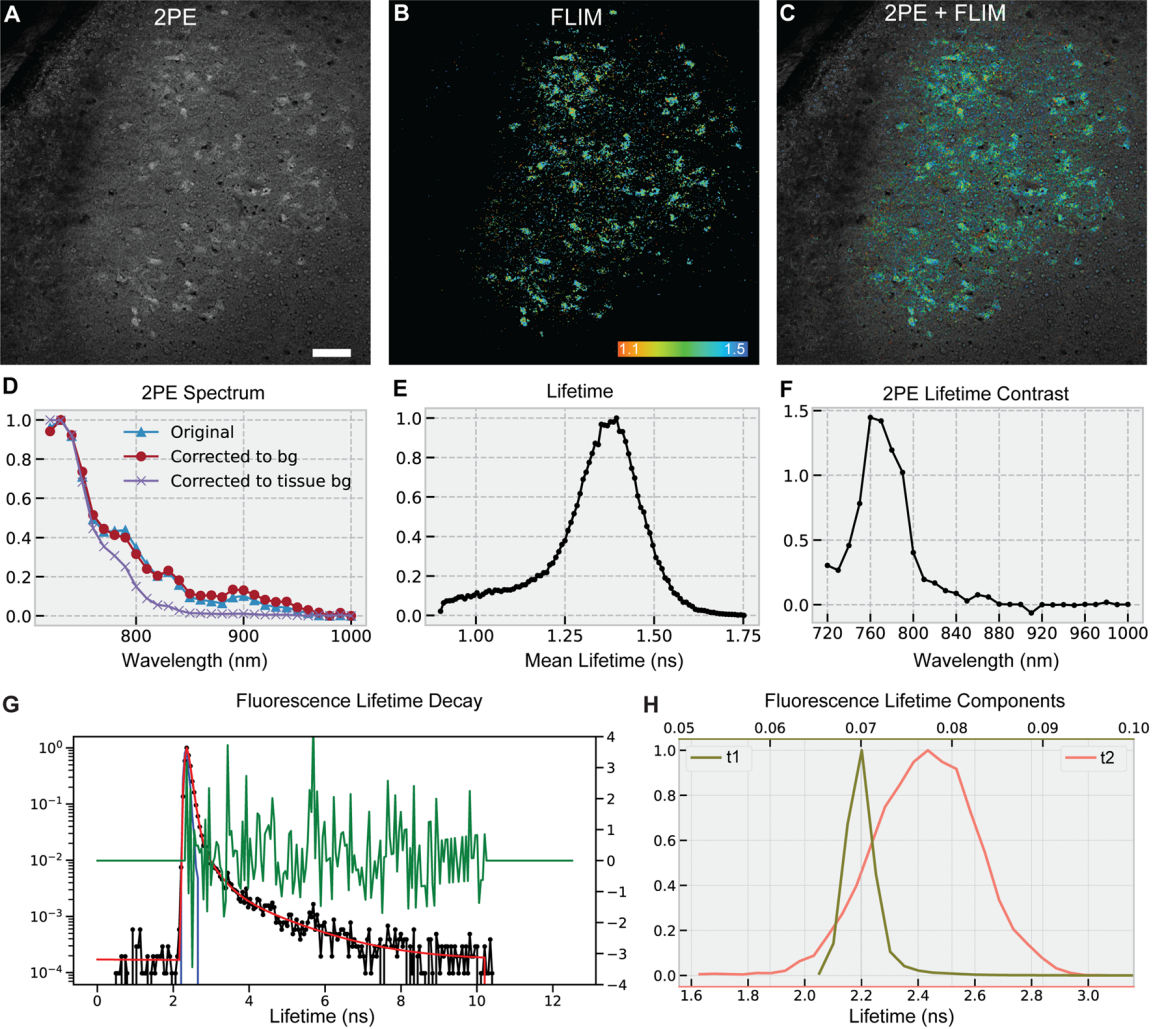
Two-photon excitation (2PE) fluorescence spectral and lifetime properties of Fluoro-Gold (FG) in tissue and aqueous solution. Fluorescence intensity was normalized based on average laser power. (**A**) 2PE fluorescence imaging of murine facial motor nucleus labeled with FG at 730 nm. (**B**) FLIM image. (**C**) FLIM image on top of raw data. Scale bar 100 μm. (**D**) 2PE absorption spectra obtained using our microscope in paraformaldehyde-fixed brainstem tissue had a maxima at 720 nm, regardless of correction for tissue autofluorescence, background tissue fluorescence, or background optical fluorescence. (**E**) The apparent lifetime distribution of FG was 1.4 ns in paraformaldehyde-fixed murine brainstem. (**F**) Maximum 2PE lifetime contrast between FG and background tissue fluorescence occurred at 760 nm. (**G**) 2PE decay of FG in aqueous solution (pH 6.5) fit to a bi-exponential decay model demonstrates two different lifetime components; t1 < 100 ps and t2 = 2.3 ns. A sample fitting curve for the solution is shown in panel (**G)**. On the left axis, the black line shows the data, red the fit, blue the IRF, and green the residuals (on the right vertical axis). (**H**) The panel shows the distribution of the biexponential fit with the distribution of two lifetimes that generate the mean lifetime of 1.4 ns. The distribution is derived from multiple pixels of the FLIM image of a drop of FG solution. The top horizontal axis shows the shorter lifetime (t1, olive-colored), and the bottom horizontal axis shows the longer lifetime (t2, salmon-colored).

**Figure 2:**
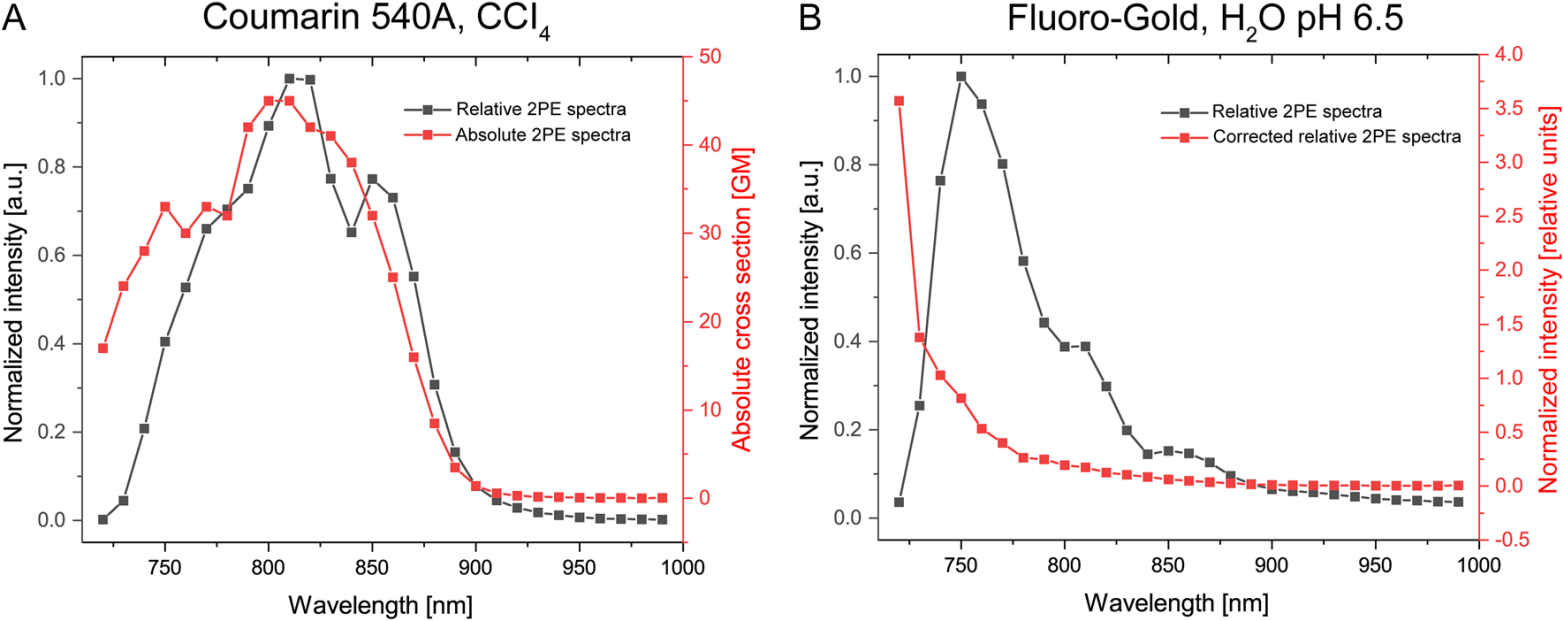
Two-photon excitation spectra of Coumarin 540A in carbon tetrachloride (CCl_4_) and Fluoro-Gold in distilled water, pH 6.5. Knowledge of absolute two-photon cross sections of Coumarin 540A enables calculation of a corrected relative 2PE spectra of FG given relative fluorescent intensities measured under identical imaging conditions.

The 2PE fluorescent spectral and decay properties for FG in aqueous solution (2% w/v in distilled water) and murine brainstem tissue fixed with a phosphate-buffered formalin solution were determined using a commercial multiphoton microscope, powered by a tunable mode-locked Ti:Sapphire laser between 720–990 nm (Fig. 1A–C). A second commercial multiphoton microscope, powered by a tunable dual-output Ytterbium laser between 720 and 1300 nm, was employed to verify the quadratic dependence of FG’s fluorescence emission (Supplementary Fig. S1). Maximal 2PE for FG in brainstem tissue (adjusted for background tissue fluorescence) occurred at 720 nm (Fig. 1D). Corrected relative 2PE spectra for FG were estimated using relative methods and confirmed maximum excitation within the tested range at 720 nm (Fig. 2). Peak one-photon absorption of FG was observed at 350 nm and peak emission at 405 nm (Supplementary Fig. S2).

The apparent fluorescence lifetime of FG-labeled facial motoneurons was measured at 1.4 ns using fitting procedures (Fig. 1E). Optimal excitation to maximize lifetime contrast between FG-labeled cell bodies and background brainstem tissue fluorescence was 760 nm (Fig. 1F). The fluorescence lifetime of FG in aqueous solution and brainstem tissue followed a bi-exponential distribution (Fig. 1G). Two components were resolved, a high-intensity fluorescent decay (a_1_) with a lifetime of less than 100 ps, and a low-intensity decay (a_2_) with a lifetime of 2.3 ns (Fig. 1H). The background lifetime was measured at 1.35 ns (averaged across all excitation wavelengths, Supplementary Fig. S3). The decay curve for FG was recorded at 740 nm (Fig. 1G, Supplementary Fig. S4). The graph demonstrated two different components and these two components were calculated using a bi-exponential fitting. The fitted curve overlapped well with the decay curve, and the residuals distributed randomly around 0. The chi-squared value of 1.34 verified a good fit.

**Figure 3:**
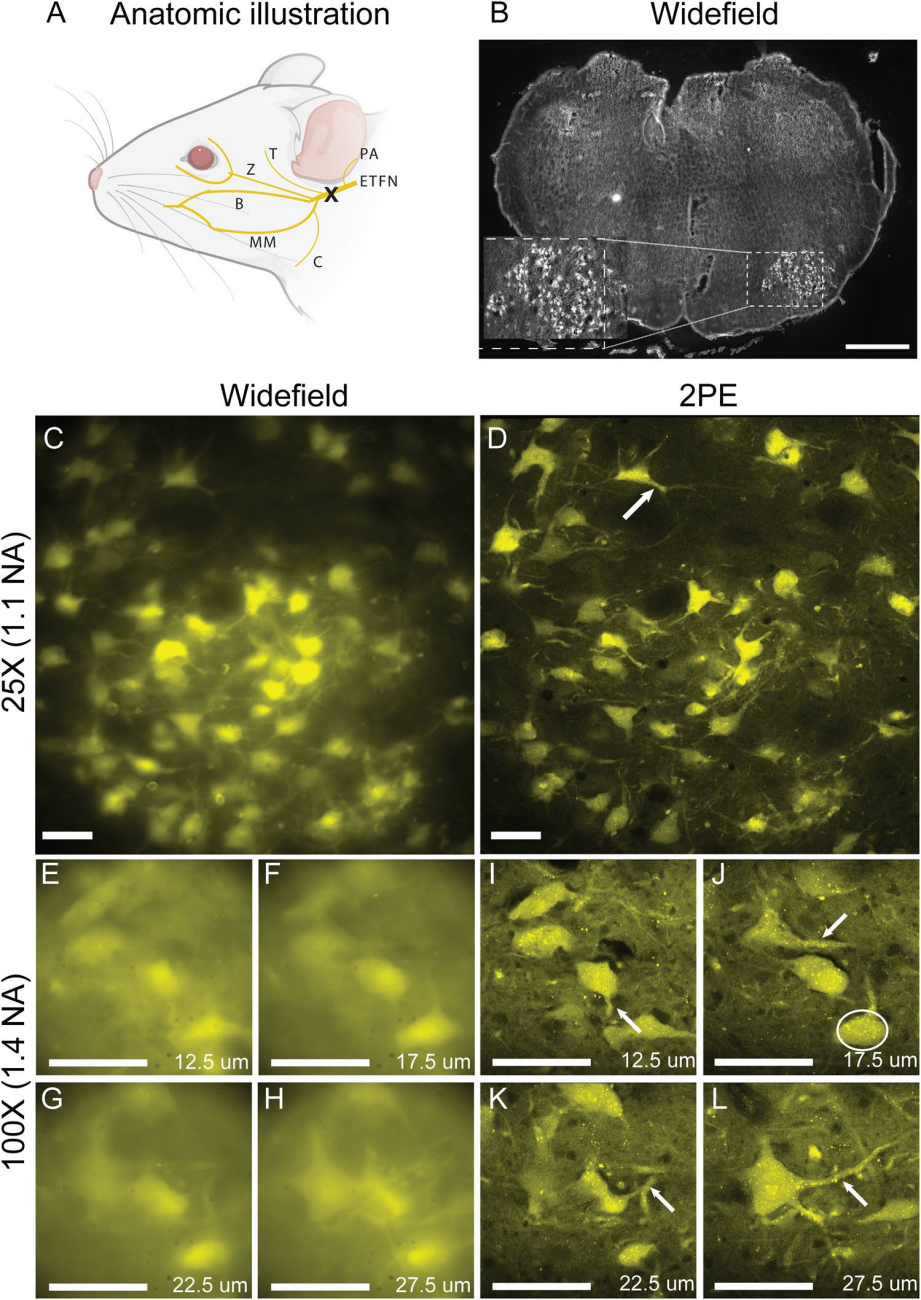
Retrograde labeling of murine facial motor nucleus with Fluoro-Gold. (**A**) The extratemporal main trunk of the facial nerve was transected distal to the auricular branches, and immersed in Fluoro-Gold for 10 min (transection site marked by X). Created with bioRender, https://biorender.com. (**B**) Visualization of the left facial motor nucleus by widefield fluorescence microscopy; robust staining of all subnuclei excluding the medial subnucleus of the auricular branches is noted. ETFN = extratemporal facial nerve. PA = postauricular branch. MM = marginal mandibular branch. C = cervical branch. B = buccal branch. Z = zygomatic branch. T = temporal branch. (**C–L**) Widefield versus two-photon excitation microscopy of Fluoro-Gold-labeled 40 µm thick murine facial motor nuclei sections. Superior image resolution and optical sectioning are noted for 2PEM at 25x (**C,D**) and 100x (**E–L**) magnification. Excellent visualization of FG accumulation in dendrites (white arrows) and punctate subcellular accumulations (white circle in panel **H**) is observed within 2PEM images. Scale bars: B, 1 mm; C-L, 50 µm.

Immersion of transected murine extratemporal facial nerve main trunk in FG for 10 min resulted in excellent visualization of intermediate, lateral, and dorsolateral facial subnuclei within 6 days (Fig. 3A,B). For multilabel experiments, immersion of transected murine facial nerve buccal and zygomatic branches in FG and Fluoro-Ruby (FR), respectively, yielded facial motor subnuclei-specific labeling (Supplementary Fig. S5).

**Figure 4:**
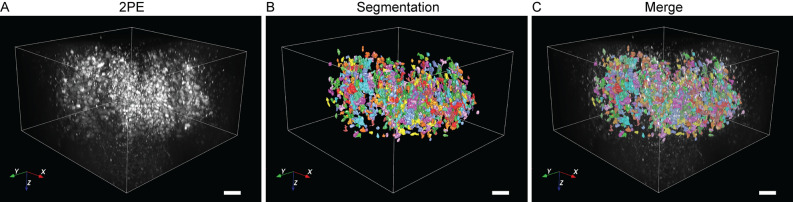
Deep two-photon excitation microscopy imaging of whole-mount Fluoro-Gold labeled murine facial motor nucleus*.* (**A**) Three-dimensional reconstruction of x–y–z tile scans. (**B**) Neuron cell body segmentation and automated cell counts demonstrating 875 labeled cell bodies. (**C**) Merged segmented and raw data. Scale bar 100 um. Reconstructions and segmentation performed using commercial software (Aivia v9.5, SVision, Bellevue, WA).

2PEM imaging of murine brainstem tissue demonstrated superior lateral and axial resolution at 25 × and 100 × magnification compared to widefield fluorescence microscopy (Fig. 3). Dendrites and subcellular structures were visualized using 2PEM, whereas these structures were poorly resolved on widefield microscopy (Fig. 3). Excellent optical sectioning and 3D reconstruction of the facial nucleus were performed on sectioned and whole-mount tissues (Figs. 3 and 4). High-resolution imaging depths in excess of 450 µm were achieved in whole-mount tissue, using refractive index matching and increasing optical power with depth to compensate for signal attenuation. Machine learning-based image segmentation was used for automated quantification of labeled cell bodies (Fig. 4).

## Discussion

The present study is the first to characterize the 2PE fluorescent spectral and lifetime characteristics of FG, a fluorescent dye considered by many as the “gold standard” for retrograde neuronal labeling^29^. FG is an excellent retrograde tracer due to its bright fluorescence, efficient labeling, lack of diffusion to neighboring cells, labeling of cell body cytoplasm and dendrites, and its resistance to fading^2^. However, direct imaging of FG-labeled samples has been limited to thin sections using widefield microscopy as the dye is poorly excited by lasers typically present on commercial confocal systems^14,28,29^. Though one prior publication employed 2PEM for imaging of FG-labeled neurons within thick tissue sections, optimal 2PE characteristics of the dye were not reported^24^.

The corrected relative 2PE spectra of FG in acqueous solution and 2% PFA fixed tissues yielded a maximum signal at 720 nm over the tested range, approximately twice its peak single-photon excitation^1,13^. Figure 2B suggests the absolute maximum of the 2PE absorption spectra occurs at a wavelength shorter than the minimum tuning range of the microscopes employed herein. The 2PE spectra of FG in murine brainstem tissue suggest that 760 nm excitation may yield optimal results to optimize signal to background ratio where squelching of background is not performed (eg. using Sudan black). We demonstrated improved image resolution and optical sectioning by means of 2PEM over conventional approaches, with near-ideal power-squared dependence (Supplementary Fig. S1)^33,41^. The achieved resolution by 2PEM was sufficient for visualization of dendrites and subcellular structures (Fig. 3). The utility of 2PEM in resolving FG-labeled dendrites sufficient for quantification carries relevance to the field of neuropathology, as dendritic number and morphology changes are associated with neurodegenerative diseases^44^. The FG-labeled subcellular structures resolved by 2PEM likely represent dye accumulation in neuronal endosomes and lysosomes (Fig. 3J)^14^.

The imaging depth of 2PEM is limited by optical aberration and light attenuation caused by absorption and scattering of light within thick biological samples^31^. Spherical aberration, which is caused by optical inhomogeneities within the sample and the immersion medium, blurs images and causes loss of detail in deep tissue^45^. Several techniques for clearing the tissue have been proposed to increase the imaging depth in fixed tissue samples. An excellent review of these techniques was published by Richardson and Lichtman^46–48^. The present work used optical clearing via simple immersion in commercially-available refractive index matching solution due to its ease of use, effectiveness, and low cost compared to other tissue clearing techniques^47,49,50^. Herein, whole-mount murine brainstem was imaged while immersed in refractive index matching solution to depths greater than 450 μm using a commercially-available glycerol-immersion objective lens (Fig. 4). In contrast, imaging depths of FG-labeled murine facial motor nuclei were limited to 150 μm using a water immersion objective lens without refractive index matching (data not shown).

Imaging of fluorescent labels in nervous tissues may be complicated by lipofuscin-associated autofluorescence. Lipofuscin demonstrates broad excitation and emission spectra, often resulting in poor signal-to-background ratio impacting fluorescence imaging quality. Though pre-treatment of nervous system specimens with sudan black or cupric sulfate may reduce background lipofuscin fluorescence, adequate quenching while maintaining suitable fluorophore fluorescence is challenging^51,52^. Herein, we observed an average fluorescence lifetime of 1.4 ns for FG, comprised of a high-intensity fluorescent decay component with a lifetime value less than 100 ps, and a low-intensity component having a lifetime of 2.3 ns. The fluorescence lifetime of lipofuscin is 1.34^53,54^. The difference between fluorescence lifetimes of FG and lipofuscin indicates they are potentially separable using FLIM, demonstrated herein by the improvement in signal-to-background ratio with FLIM of FG-labeled murine brainstem (Fig. 1A,B).

Herein is presented the relative corrected 2PE spectra of FG between 720 and 990 nm. Determination of absolute fluorescence cross sections from relative methods requires further corrections for the spectral sensitivity of the detector and for differences in fluorescence quantum efficiencies of the tested and reference dyes. Future work will seek to characterize the absolute two-photon absorption spectrum of FG over a broader wavelength range. TPE spectra and lifetimes for other common retrograde neuronal tracers including Fluoro-Ruby should be established to facilitate multi-label studies. Estimation of the corrected relative TPE spectra of previously uncharacterized dyes is straightforward by measuring relative fluorescence cross-sections of the dye along with a known dye under identical imaging conditions^33,55^. Multilabel retrograde neuronal tracer experiments using 2PEM with FLIM for deep multicolor volumetric imaging could facilitate study of the somatotopic organization of neuronal circuits^56,57^. Protocols for 2PEM of FG in tissues cleared by solvents could be developed. FG is compatible with lipid-clearing techniques, which can extend imaging depth to span the entire thickness of the mouse cortex^25^. The present study utilized image analysis software for segmentation and automated counts of facial neuron cell bodies (Fig. 4). Prior studies have sectioned brainstem tissue and manually counted labeled cell bodies in the murine facial nucleus; a time- and resource-intensive method^9,58^. Future studies should evaluate the accuracy of automated cell body counts in volumetric imaging against tissue sectioning and manual cell counts.

## Materials and methods

### Materials

FG (Fluorochrome, Denver, CO) was used for widefield, 2PEM, and FLIM. A concentration of 2% FG (w/v in distilled water, pH 6.5) was used to minimize neurotoxicity, and employed for animal experiments and characterization of dye properties in acqueous solution. Fluoro-Ruby (10% w/v in distilled water, Invitrogen, Carlsbad, CA) was used for facial subnuclei labeling to demonstrate the utility of multi-labeling techniques incorporating FG. Tissue sections were mounted in Fluoromount-G (SouthernBiotech, Birmingham, AL). EasyIndex (Life Canvas Technologies, Cambridge, MA) was used for refractive index matching.

### Animal surgeries

Two female C57 adult mice (7 weeks) and one 4-week old female Lewis rat were used in the present study. All animal surgeries were performed in accordance with the National Institutes of Health Guide for the Care and Use of Laboratory Animals, and with approval by the Massachusetts Eye and Ear Animal Care Committee (ACC Protocol # 16-006, IRBNet ID 884247). This study was carried out in compliance with the ARRIVE guidelines. Five uL of 2% FG was delivered to the transected main facial nerve trunk of the C57 mice via the conduit reservoir technique (Fig. 3A). In the Lewis rat, 5 µL FG was delivered to the transected facial nerve buccal branch, and 5 uL 10% Fluoro-Ruby was delivered to the transected zygomatic branch to demonstrate labeling of facial subnuclei.

Procedures were performed under isoflurane anesthesia, 1–3% maintenance dosing in 1 L/min O_2_. Buprenorphine (0.05 mg/kg subcutaneously) and meloxicam (1.0 mg/kg subcutaneously) were administered prior to skin incision. A 1 cm infra-auricular incision was made on the left, and skin-muscle flaps were elevated. The exorbital lacrimal gland was retracted to expose the proximal pes anserinus and main trunk. The main trunk was meticulously dissected circumferentially using the operating microscope at 25 × magnification. Care was taken to prevent crush injury. The nerve was transected and the proximal stump was immersed in a pipette tip containing 5 µL of dye solution for 10 min. The wound bed was irrigated with saline and closed in a single layer using 4–0 absorbable suture (Polysyn, Sharpoint, Westwood, MA). Animals were recovered from general anesthesia and returned to their cages. Postoperative meloxicam was given for 72 h post-procedure. The identical procedure was performed for the Lewis rat, except the buccal and zygomatic facial nerve branches were transected for delivery of FG and FR, respectively.

### Tissue harvest

After a six-day survival period, animals underwent CO_2_ euthanasia and cardiac perfusion using 2% phosphate-buffered paraformaldehyde fixative (PFA) solution. Animal heads were placed in 2% PFA overnight, then underwent brainstem harvest at the level of the facial nucleus. The intracranial facial nerve was used as a landmark for facial motor nucleus identification. Specimens were cryo-protected in 30% sucrose for 24 h prior to embedding in optimal cutting temperature (OCT) compound and coronal cryosectioning at 40 µm (Leica CM3050 S, Leica Biosystems, Buffalo Grove, IL). Slides were mounted using Fluoromount-G to minimize photobleaching during subsequent imaging. Whole-mount brainstems were imaged following overnight fixation in 2% PFA, followed by rinsing in phosphate buffered saline, and six-hour incubation in refractive-index mataching solution (EasyIndex, LifeCanvas Technologies, Cambridge, MA) on a shaker at room temperature.

### Fluoro-Gold spectral characterization

A Horiba FluoroMax-4 spectrophotometer (Horiba Instruments, Japan) was used to collect one-photon absorption and fluorescent emissions of FG in aqueous solution (2% w/v in distilled water).

Two-photon excitation fluorescent spectral properties of FG were first characterized in dilute solution (3.76E−2M) to avoid wavelength-dependent absorption and scattering. To avoid need for tedious characterization of and correction for laser properties in calculating the absolute fluorescence cross-sections, comparative methods may be alternately employed^59^. Herein, corrected relative 2PE spectra of FG, expressed in relative units, were estimated by a relative fluorescence method using Coumarin 540A (Exciton, 05450) as a reference standard. Coumarin 540A was dissolved in carbon tetrachloride (CCl_4_, 3.76E−4M) and relative 2PE spectra were calculated^59^. Its relative 2PE spectra were compared with its absolute spectral shape from published data to obtain a correction curve^60^.

$${f}_{c}\left(\lambda \right)=\frac{{F}_{R}(\lambda )}{{\sigma }_{R}(\lambda )}$$

This correction function was then applied to the relative 2PE spectrum of FG and imaged under identical experimental conditions to get its relative 2PE spectra.

$${\sigma }_{S}(\lambda )=\frac{{F}_{S}(\lambda )}{{f}_{c}\left(\lambda \right)}\frac{{C}_{R}}{{C}_{S}}$$

The relative 2PE spectra were corrected by the ratio of molar concentrations of Coumarin 540A and FG.

### Tissue imaging

Comparison of widefield and 2PEM imaging was performed on a commercial multiphoton microscope (TrimScope II, LaVision Biotech) powered by a dual-output femtosecond linear horizontal polarized laser (Insight X3, SpectraPhysics) at 760 nm. Images were acquired with a set of galvanometer mirrors and piezo XYZ-stage for large-field volumetric imaging. For thick sections, a water-immersion objective lens with correction collar (N25X-APO-MP1300, WD 2.0 mm, Nikon). For whole-mount imaging, optical clearing was achieved by imaging tissue in refractive index matching solution (EasyIndex) using a glycerol-immersion objective lens (CLr Plan-Neofluar 20x, WD 5.6 mm, Carl Zeiss). A long-pass filter was employed to separate excitation and emission light (T680lpxxr, Chroma Technology Corp, Bellows Falls, VT, USA), and a second dichroic mirror at 435 nm was used to split the emission light in the non-descanned detection path. Fluorescent signal was filtered (Semrock 525/50 nm) and collected using a GaAsP photomultiplier tube (H 7422-40, Hamamatsu). Image acquisition was averaged four times to improve signal-to-noise ratio. The quadratic dependence of 2PE fluorescence was assessed from fluorescent images by measuring the variation of fluorescence intensity with increasing excitation intensity. Widefield epifluorescent imaging was performed using the multiphoton microscope stage, broadband light-emitting diode excitation (pE-300 ultra, CoolLED, Andover, England), and a monochrome Zyla 4.2 sCMOS camera (Andor Technology Ltd, Belfast, Northern Ireland). Microscope control was achieved via open-source software (LaVision BioTec ImSpector Software).

FLIM data was acquired using an upright multiphoton microscope (Ultima, Bruker Microscopy, Middleton, WI) coupled to an ultrafast tunable NIR linear horizontal polarized laser (Insight DS, Spectra-Physics, CA) between 720 and 1100 nm. A 700 nm short-pass filter (ET700SP-2P8, Chroma Technology Corp.) was employed to separate excitation and emission light, and a second filter at 460 nm (FF02-460/80-25, Semrock, USA) used to split the emission light in the non-descanned detection path. Images were collected using a 10X/0.5NA objective lens (CFI Super Fluor, Nikon, WD 1.1 mm). No correction was made for spectral properties of the lens, however measurements were repeated using two additional 2PEM imaging objective lenses (20x/0.75 Nikon, CFI Apochromat LWD Lambda S 20XC W.I., and CFI Plan Apochromat Lambda S 10X), and no significant differences in the FG excitation spectrum were observed. Optical power was continuously measured during experiments using a silicon-based photodetector power meter (918D-SL-OD3R, Newport, MKS, CA), digitized using an open-source microcontroller (Arduino UNO, Arduino, Ivrea, Italy), and the digital laser power value was added to the metadata of the file using PrairieView software (Bruker, Billerica, MA). Fluorescence intensity and lifetime distribution analyses were performed on dye solution (2% W/V in distilled water) and FG-labeled brainstem sections. The decay curve for FG was recorded at 740 nm (Supplementary Fig. S5). The graph demonstrated two different components and these two components were calculated using a bi-exponential fitting. The fitted curve overlaps well with the decay curve, and the residuals distribute randomly around 0. The chi-squared values for all the fits presented in this work ranged between 0.9 and 2.0. Fitting routines were also aided with visual analysis of the residual curves, which demonstrated minimal variations. The instrumental response function of the system had a duration of 100 ps (Supplementary Fig. S6).

Dye solutions were loaded into sealed glass capillary tubes and imaged over 10 nm wavelength intervals between 720 and 1000 nm. Fast-electronics comprising a hybrid detector (HPM-100-40, Becker&Hickl, Berlin, Germany) and time-correlation single-photon counting card (SPC-150, Becker&Hickl, Berlin, Germany) encoded photon arrivals times. Timing data of individual photons were binned and mathematically fit to double-exponential curves using commercial software (SPCImage, Becker&Hickl, Berlin, Germany). Resultant lifetime and intensity analyses and spectral plots for specific wavelengths were performed using ImageJ^61^.

### Image processing

Facial motor neuron cell body counts were quantified from original 3D data sets using commercial machine learning software (Aivia v9.5, SVision Technologies, Bellevue, WA). A random forest pixel classifier was trained by painting examples of cell body signal and background signal^62^. This classifier was used to generate a signal channel in Aivia’s 3D object mesh recipe consol to highlight and segment neuronal cell bodies. The object meshes were generated in a region of interest and adjusted in an iterative manner, during which morphological smoothing was performed, minimum object edge intensity defined, and holes filled until a satisfactory result was obtained. The segmentation parameters were then applied to the entire volume to generate a channel highlighting pixels comprising cell bodies.

## Supplementary Information

Supplementary Information.
