# Supplementary material for: Two-photon excitation fluorescent spectral and decay properties of retrograde neuronal tracer Fluoro-Gold
Source: Sci Rep. 2021 Sep 10;11:18053. doi: 10.1038/s41598-021-97562-3 (PMC8433443; doi:10.1038/s41598-021-97562-3)
Supplement: Supplementary file 1 — Supplementary Information. [file 41598_2021_97562_MOESM1_ESM.docx]

**Supplementary Information**

**Two-photon excitation fluorescent spectral and decay properties of retrograde neuronal tracer Fluoro-Gold**

Matthew Q. Miller^1,3,#^, Iván Coto Hernández^1,#,*^, Jenu V. Chacko^2^, Steven Minderler^1^, Nate Jowett^1,*^

^1^Surgical Photonics and Engineering Laboratory, Massachusetts Eye and Ear, Harvard Medical School, Boston, Massachusetts

^2^Laboratory for Optical and Computational Instrumentation, University of Wisconsin, Madison, WI, United States

^3^Department of Otolaryngology/Head and Neck Surgery, University of North Carolina Health Care, Chapel Hill, North Carolina, USA.

^#^ These authors have contributed equally to the work.

^*^ Corresponding author: [ivan_cotohernandez@meei.harvard.edu](mailto:ivan_cotohernandez@meei.harvard.edu); [nate_jowett@meei.harvard.edu](mailto:nate_jowett@meei.harvard.edu)


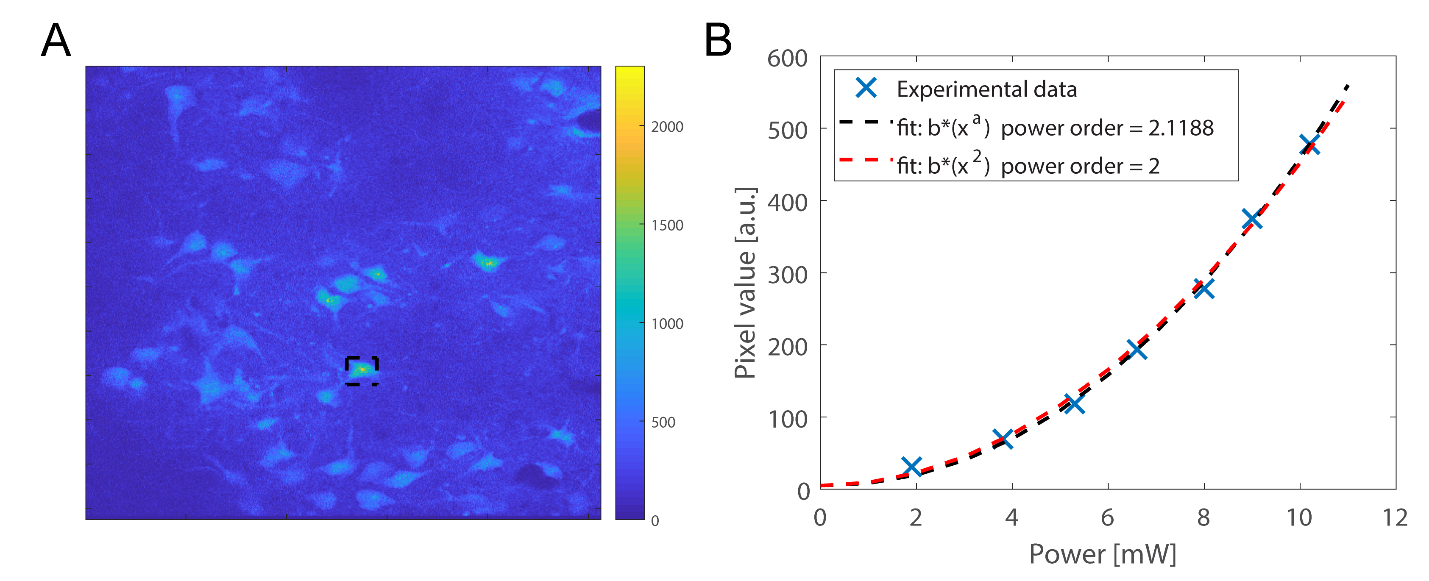


**Supplementary Figure S1.** Power dependence of 2-photon excitation (2PE) fluorescence signal of Fluoro-Gold labelled murine facial motor nucleus. (A) 2PE imaging of FG-labeled motor neuron cell bodies at 760 nm (arbitrary unit (a.u.) in color bar). (B) The fluorescent emission signal of Fluoro-Gold demonstrates quadratic dependence on excitation power. Laser average power was measured before passing through the objective lens.


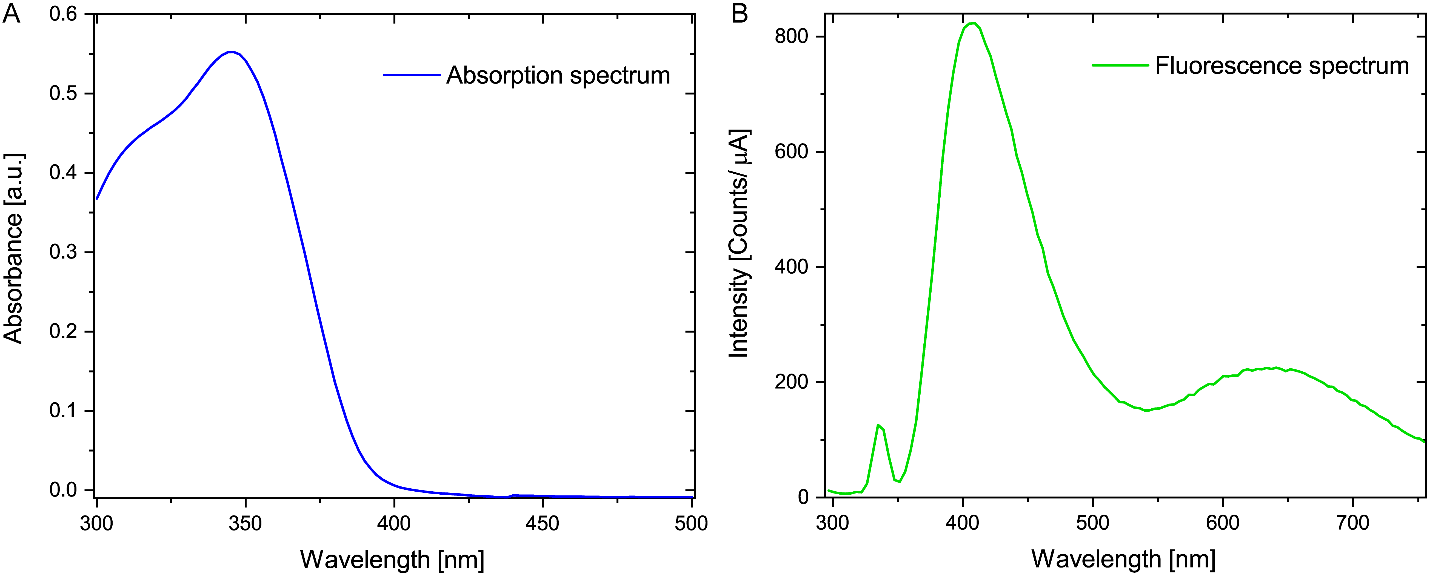


**Supplementary Figure S2.** One-photon absorption and fluorescence spectra of Fluoro-Gold in distilled water, pH 6.5. Fluoro-Gold has a maximum absorption at 350 nm and a broad emission band with a peak at 405 nm.


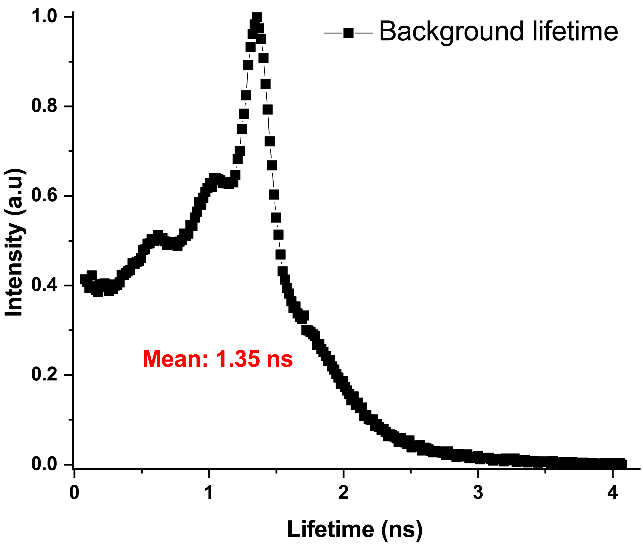

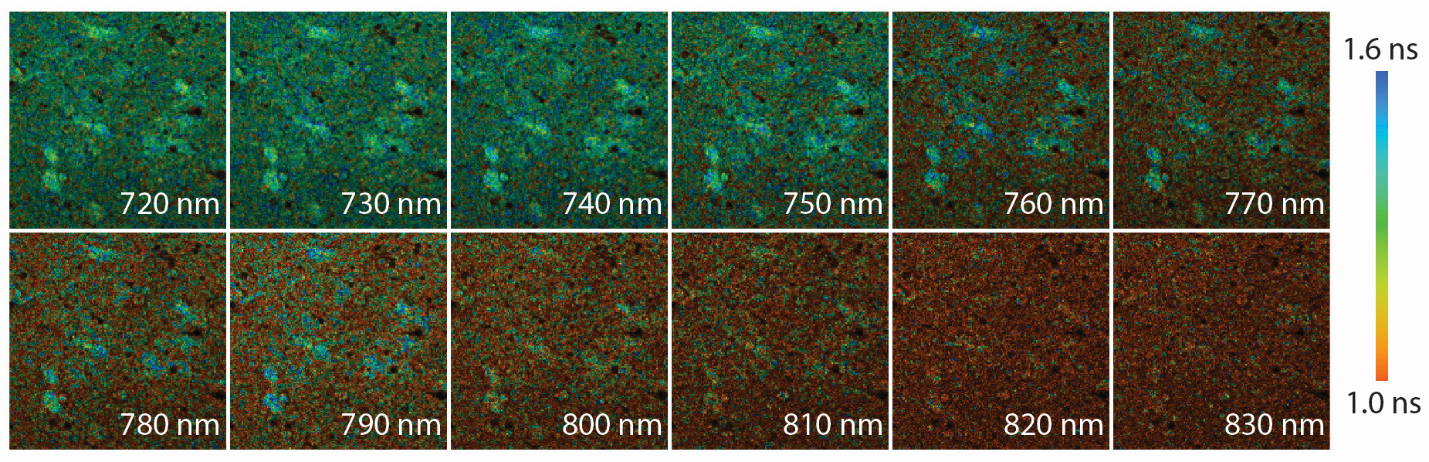


**Supplementary Figure S3.** Background mean fluorescence lifetime of Fluoro-Gold labelled phosphate-buffered paraformaldehyde-fixed murine brainstem tissue. The mean lifetime observed across excitation wavelengths between 720 nm and 780 nm was 1.35 ns. Above 800 nm, background intensity was minimal and not used in the distribution shown above.

**
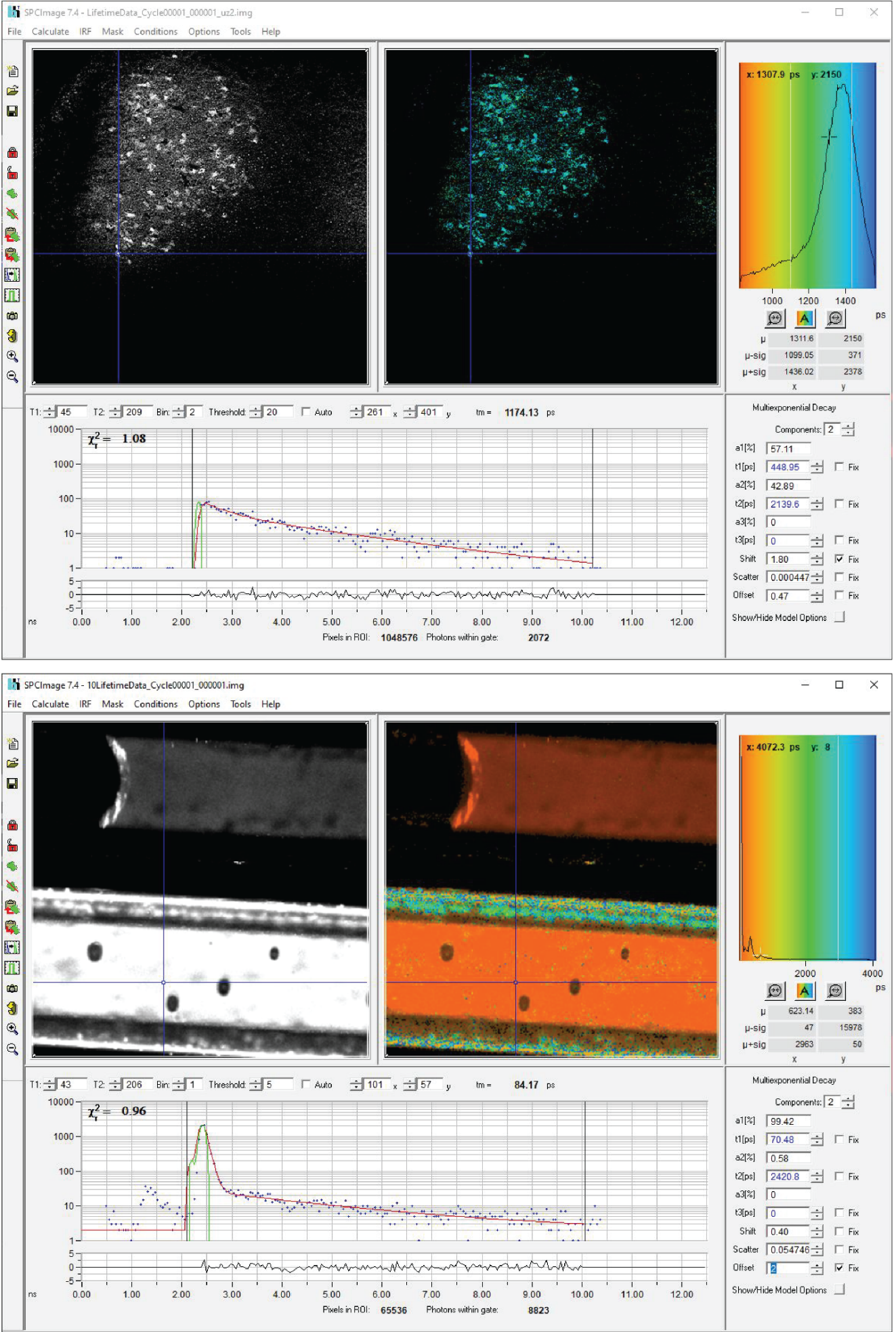
**

**Supplementary Figure S4.** Decay curve for Fluoro-Gold in aqueous solution and phosphate-buffered paraformaldehyde fixed murine brainstem tissue at an excitation of 740 nm. (Top) 2PE imaging of FG-labeled cell bodies in the murine facial motor nucleus. (Bottom) 2PE imaging of FG in distilled water, pH 6.5. Graphs demonstrated two different components, calculated using a bi-exponential fitting. Fitted curves (red lines) overlap well with raw decay data (blue dots), with residuals distributed randomly around 0 (green line). Chi-squared values near one verified a good fit.

**
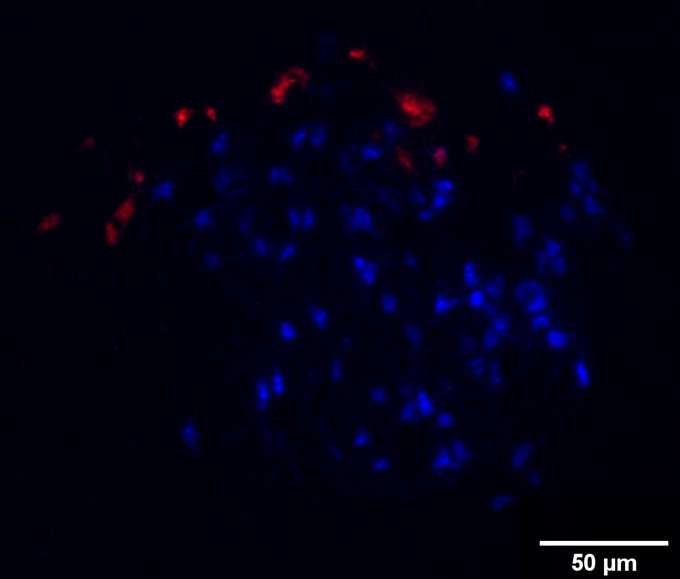
**

**Supplementary Figure S5.** Widefield microscopy (5x) image of rat facial nucleus with targeted double-labelling using Fluoro-Gold and Fluoro-Ruby. Fluoro-Gold was delivered to the transected facial nerve buccal branch and Fluoro-Ruby was delivered to the transected zygomatic branch using conduit reservoir technique six days prior to tissue harvest. Targeted labelling of lateral and intermediate subnuclei (blue, Fluoro-Gold) and dorsolateral subnucleus (red, Fluoro-Ruby) is demonstrated.

**Supplementary Figure S6.** Instrumental response function for decay measurements. The calculated instrumental response function was 100 ps. This was measured using SHG signal from a Urea crystal at 740nm. The IRF was measured for different wavelengths and applied to fits in the range of +/- 10 nms to compensate for the shift in the fitting due to change in wavelength. For example, an 800nm SHG of Urea was used as the IRF for excitation range 795-805nm.
